# Steroidogenic control of liver metabolism through a nuclear receptor-network

**DOI:** 10.1016/j.molmet.2019.09.007

**Published:** 2019-09-30

**Authors:** Alexandra Milona, Vittoria Massafra, Harmjan Vos, Jyoti Naik, Natalia Artigas, Helen A.B. Paterson, Ingrid T.G.W. Bijsmans, Ellen C.L. Willemsen, Jose M. Ramos Pittol, Irene Miguel-Aliaga, Piter Bosma, Boudewijn M.T. Burgering, Catherine Williamson, Santiago Vernia, Waljit S. Dhillo, Saskia W.C. van Mil, Bryn M. Owen

**Affiliations:** 1 MRC London Institute of Medical Sciences (LMS), London, United Kingdom; 2 Institute of Clinical Sciences (ICS), Faculty of Medicine, Imperial College London, London, United Kingdom; 3 Center for Molecular Medicine, University Medical Center Utrecht and Utrecht University, Utrecht, the Netherlands; 4 Amsterdam UMC, University of Amsterdam, Tytgat Institute for Liver and Intestinal Research, AG&M, Meibergdreef 69-71, 1105 BK, Amsterdam, the Netherlands; 5 School of Life Course Science, Kings College London, London, United Kingdom; 6 Section of Endocrinology & Investigative Medicine, Division of Diabetes, Endocrinology, and Metabolism, Department of Metabolism, Digestion, and Reproduction, Imperial College London, London, United Kingdom

**Keywords:** FXR, FGF21, Bile acids, Liver, Metabolism, Fasting, Gluconeogenesis, Diabetes, Steroidogenesis, Cyp17a1, DHEA, Dehydroepiandrosterone, PPARα, Peroxisome proliferator-activated receptor alpha, FXR, Farnesoid X receptor, SHP, Small heterodimer partner, LRH-1, Liver receptor homologue 1

## Abstract

**Objective:**

Coupling metabolic and reproductive pathways is essential for the survival of species. However, the functions of steroidogenic enzymes expressed in metabolic tissues are largely unknown.

**Methods and results:**

Here, we show that in the liver, the classical steroidogenic enzyme Cyp17a1 forms an essential nexus for glucose and ketone metabolism during feed-fast cycles. Both gain- and loss-of-function approaches are used to show that hepatic Cyp17a1 is induced by fasting, catalyzes the production of at least one hormone-ligand (DHEA) for the nuclear receptor PPARα, and is ultimately required for maintaining euglycemia and ketogenesis during nutrient deprivation. The feedback-loop that terminates Cyp17a1-PPARα activity, and re-establishes anabolic liver metabolism during re-feeding is mapped to postprandial bile acid-signaling, involving the receptors FXR, SHP and LRH-1.

**Conclusions:**

Together, these findings represent a novel paradigm of homeostatic control in which nutritional cues feed-forward on to metabolic pathways by influencing extragonadal steroidogenesis.

## Highlights

• The classical steroidogenic enzyme, Cyp17a1, is upregulated in liver during fasting.
• CYP17a1 produces a hormone-ligand for the nuclear receptor PPARα and affects glucose and lipid handling in the liver.
• Hepatic Cyp17a1 is essential for maintaining glycaemia and ketones during fasting.
• Bile acids, via a nuclear receptor cascade, repress hepatic *Cyp17a1* as part of the re-feeding response.

## 1 Introduction

Metabolic and reproductive pathways are intimately linked. In mammals, nutritional cues govern fertility, at least in part, by modulating the activity of classical steroidogenic enzymes in the gonads [1]. However, many of these enzymes are also expressed in metabolic tissues [2]. One example is the cytochrome P450 enzyme Cyp17a1, which has both 17α-hydroxylase activity and 17,20-lyase activity. It catalyzes intermediate reactions in the synthesis of all steroid hormones, via the intermediate dehydroepiandrosterone (DHEA) [3]. Cyp17a1 is also expressed in organs that are considered non-steroidogenic, including the liver, where its function is largely unknown [4,5].

Metabolic regulation by the liver is under the control of extra-hepatic hormones and nutrient-sensing members of the nuclear receptor class of transcription factors. For example, the PPARα is activated by circulating free fatty acids and is considered a master regulator of the adaptive starvation response [[6], [7], [8], [9]]. By contrast, the FXR is activated postprandially by bile acids returning to the liver from the intestine [6]. As such, FXR and PPARα can directly drive expression of genes involved in anabolic and catabolic processes, respectively, in the liver. However, FXR can also act as a trans-repressor of gene expression. In the bile-acid synthesis pathway, for example, FXR induces the expression of the small heterodimer partner (SHP), which represses expression of Cyp7a1 by preventing binding of the liver receptor homologue 1 (LRH1) to its promoter [10]. Intriguingly, FXR is also expressed in steroidogenic cells of the gonads [11], many of its target-genes are P450-enzymes, and bile acids themselves are steroid-like molecules that provide feedback on its activity [12]. As such, FXR may potentially represent a previously unexplored integrator of metabolic and steroidogenic processes.

Here, we have discovered that the steroidogenic enzyme Cyp17a1 is repressed by FXR-action in the liver in the fed-state. However, during starvation, Cyp17a1 is de-repressed and produces a hormone-ligand (DHEA) for PPARα. We show that hepatic Cyp17a1-dependent PPARα-activity is essential for the maintenance of fasting glucose and ketone levels. As such, this axis represents an important new link between extra-gonadal steroidogenic-pathways and a nutrient-responsive nuclear receptor network in a metabolic tissue.

## 2 Materials and methods

### 2.1 Animal experiments

Experiments were approved by the ethics committee of the University Medical Center Utrecht and were in accordance with European law 9–12 or performed in accordance with the UK Animals (Scientific Procedures) Act 1986. All animals were housed in a room with controlled temperature (20–24 °C), a 12 h light dark cycle, and free access to food and water.

### 2.2 Plasmid constructs

Approximately 2.5 kb of the human *CYP17A1* promoter was amplified by PCR using the following primer pair: human, 5′-gatcggtaccATAGCACACCATATTCCTAC-3’ (sense); and human, 5′-gatcgctagcGTAAGCAGCAAGAGAGCCACG-3’ (antisense). The resultant fragment was inserted into the *Kpn1* and *Nhe1* site of pGL3-Basic, a promoter-less luciferase reporter vector (Promega, Madison, WI). The pGL3-ratCYP7A1 promoter construct and pCMX-hSHP [10] were provided by Prof Steve Kliewer (UT Southwestern Medical Center, Dallas, TX). The pBABE-hLRH1 plasmid was a gift from Dr Mark Christian (Imperial College, London).

### 2.3 AAV injections

AAV8 particles (5x10e12gc/kg) produced according to standard protocols and quantified by qPCR were injected in to the tail-vein of mice. The constructs *shCyp17a1*, *shControl* contained a short hairpin towards Cyp17a1 or a scrambled control behind the H1 promoter. In *Cyp17a1*, and *GFP* the transgene expression was driven off the liver-specific LP1-promotor.

### 2.4 RNA isolation, cDNA synthesis and RT-qPCR

Total RNA of mouse tissue was isolated using TRIzol reagent (Invitrogen, Carlsbad, CA). RNA was reverse transcribed using the iScript cDNA Synthesis Kit (Bio-Rad Laboratories, Hercules, CA). Real-time PCR was carried out on a MyIQ real time PCR thermal cycler (Bio-Rad). mRNA expression of genes of interest was normalized to *Cyclophillin*. Primers sequences are as published [13], purchased from Sigma Aldrich, or available on request.

### 2.5 Immunoblotting

Liver tissue extracts were extracted and protein concentration was assessed (Thermo Scientific, Waltham, MA). Western blots were probed with antibodies against Cyp17a1 (1:500, sc-46081, Santa Cruz Biotechnology, Dallas, TX) and α-actin (1:5000, ab8224, Abcam, Cambridge, UK). Immunoreactivity was detected with horseradish peroxidase-conjugated antibodies and chemiluminescence (DAKO, Agilent Technologies, Santa Carla, CA).

### 2.6 Electro mobility shift assays

LRH-1 protein was *in vitro* translated using the TNT Quick Coupled Transcription/translation system (Promega). Binding reactions contained 25 mM Hepes pH 7.9, 1 mM EDTA, 0,5 mM EGTA, 5% glycerol, 1% NP-40 50 mM NaCl, 20mMDTT, 1 μg of poly (dI-dC), and 2 μl *in vitro* translated LRH-1. Samples were pre-incubated at room temperature for 5 min prior to the addition of ^32^P-labeled double-stranded oligonucleotide probes as indicated. Where indicated, LRH-1 antibody or cold specific or non-specific (CYP17a1 or Cyp17a1 RE) probes were added to the pre-incubation mix at a 12-fold molar excess. Samples were held at room temperature for further 30 min, and the protein-DNA complexes were resolved on a pre-electrophoresed 5% polyacrylamide gel in 0.5 × TBE. [^32^P]-labelled probes were detected by autoradiography.

### 2.7 Chromatin immuno precipitation

30 mg of snap-frozen liver tissue from WT mice was cross-linked using DSG and formaldehyde, as described [14]. The nuclei were extracted and sonicated to yield 500–1,000 base-pair (bp) DNA fragments. ChIP was performed like previously described [15], using an anti-LRH1 antibody (PP-H2325-00, R&D Systems, Minneapolis, MN) or IgG as control. Primer sequences are available on request.

### 2.8 Reporter assays

HEK293T cells were grown in 96-multiwell plates and co-transfected with empty pGL3-IBABP, pGL3-Cyp17a1, CMV-Renilla and either empty vector, pCMX-hSHP, or pBABE-hLRH-1 in presence or absence of different amounts of pCMX-hSHP using calcium phosphate. After 24 h, cells were incubated with DMSO and GW4064 as indicated. Cells were lysed after 24 h, and Firefly and Renilla luciferase activity were measured according to manufacturer's instructions (Promega) with a Centro LB 960 luminometer (Berthold Technologies, Chollerstr, Switzerland).

### 2.9 Plasma and liver extract analyses

Serum samples were assayed by the UVDL (University Veterinary Diagnostic Laboratory, Utrecht University), or by ELISA (b-hydroxybutyrate (Sigma)), or colorimetric assay (glucose (Sigma)). For liver extracts, 100-mg piece were weighed and homogenized in a 3:2 mixture of ethyl acetate to hexane. The organic layer was removed after over-night incubation and centrifugation, and evaporated. The residue was resuspended in PBS containing 5 mg/ml BSA and assayed for 17-OHP or DHEA by ELISA.

### 2.10 Mass spectrometry and Data Analysis

Liver protein extracts were generated by homogenizing 50 mg liver tissue in PBS and subsequent lysis in Lysis Buffer (1% NP40, 150 mM NaCl, 1 mM DTT, 50 mM Tris pH 8.0, Proteinase inhibitors (Roche, Basel, Switzerland)). 100 μg protein extract from Wt or FXR^−/-^ mice (‘light’) were mixed 1:1 with a spike-in protein extract generated from ^13^C_6_-lysine metabolically labelled mouse liver (‘heavy’) (Silantes, Munich, Germany). Proteins were denatured in urea, alkylated with iodoacetamide (Sigma–Aldrich, S Louis, MO), and digested with 1 μg of trypsin (Promega) using a Filtered Aided Sample Purification Protocol [16]. After trypsinization, peptides were fractionated based on their pH using Strong Anionic Exchange Chromatography, desalted, and acidified on a C-18 cartridge (3M, St. Paul, MN). C18-stagetips were activated with methanol and washed with buffer containing 0.5% formic acid in 80% ACN (buffer B) and then with 0.5% formic acid (buffer A). After loading of the digested sample, stagetips were washed with buffer A and peptides were eluted with buffer B, dried in a SpeedVac, and dissolved in buffer A. Peptides were separated on a 30 cm column (75 μm ID fused silica capillary with emitter tip (New Objective, Woburn, MA)) packed with 3 μm aquapur gold C-18 material (Dr Maisch, Ammerbuch-Entringen, Germany) using a 4-hour gradient (buffer A to buffer B), and delivered by an easy-nHPLC (Thermo Scientific). Peptides were electro-sprayed directly into a LTQ-Verlos-Orbitrap (Thermo Scientific) and analyzed in data-dependent mode with the resolution of the full scan set at 60000, after which the top 10 peaks were selected for CID fragmentation in the ion trap with a target setting of 5000 ions. Raw files were analyzed with Maxquant software version 1.5.1.0 [17]. For identification, the mouse Uniprot 2012 database was searched with peptide and protein false discovery rates set to 1%. The SILAC quantification algorithm was used in combination with the ‘match between runs’ tool (option set at two minutes), the IBAQ and the LFQ algorithms [18,19]. Proteins identified with two or more unique peptides were filtered for reverse hits, decoy hits and standard contaminants using Perseus software 1.3.0.4 [20]. Normalized ratios were used to quantify protein expression and further processed for comparative analysis of differential expression among the experimental conditions. Pathway and ontology analyses were performed by Ingenuity Pathway Analysis (IPA) (Qiagen, Hilden, Germany). A fold change greater than 1.3 between groups was used to select proteins as input for Ingenuity pathway analysis. Statistical significance of pathway enrichment and upstream regulator analyses were assessed by using IPA software. For the upstream regulator analysis, p-value measures whether there is a statistically significant overlap between the dataset genes and the genes that are regulated by a transcription factor/hormone/compound, based on the published data included in Ingenuity database. The mass spectrometry data have been deposited to the PRIDE Archive - proteomics data repository.

### 2.11 Statistics

See Mass Spectrometry and Data Analysis for statistical methods relating to proteomics. In all other cases a student's t-test with multiple test correction, as appropriate, was used to determine differences between groups with a p-value <0.05 selected as statistically significant.

## 3 Results

### 3.1 Hepatic *Cyp17a1* expression is regulated by feed-fast cycles via bile-acid:FXR signaling

**Figure 1:**
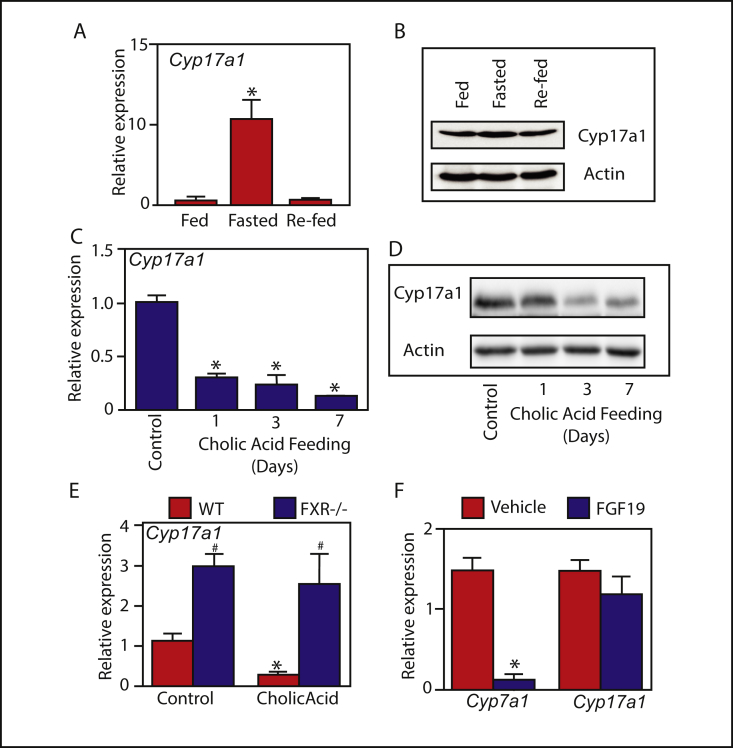
**Bile acid signaling regulates hepatic Cyp17a1 expression during feed-fast cycles.** (A and B) Hepatic *Cyp17a1* mRNA and protein expression in response to fasting (16 h) and re-feeding (6 h). (C and D) Cyp17a1 mRNA and protein expression in mice fed a control diet or a diet supplemented with 0.5% cholic acid for the indicated number of days. (E) Hepatic Cyp17a1 expression in wild-type and FXR^−/-^ mice fed a control diet or a 0.5% cholic acid-supplemented diet for 7 days. (F) Hepatic Cyp7a1 and Cyp17a1 mRNA expression in mice injected with vehicle or recombinant FGF19. N = 5–6. *p < 0.05 compared to control. #p < 0.05 compared to WT.

Expression of the steroidogenic enzyme, *Cyp17a1,* is dramatically regulated by feed-fast cycles in the liver. Over-night fasting induced hepatic *Cyp17a1* over 10-fold, while six-hours of re-feeding repressed hepatic *Cyp17a1* back to ad-libitum fed levels (Figure 1A and B). We therefore aimed to investigate the transcriptional regulation, and functional significance, of *Cyp17a1* in the liver during feeding and fasting.

Bile acids, which return to the liver postprandially, have been suggested to represent the fed-state signal responsible for repressing anabolic liver metabolism [21,22]. Therefore, we hypothesized that bile acids may be responsible for repressing hepatic *Cyp17a1* in the fed-state. This was initially tested by conducting a time-course experiment in mice fed a diet supplemented with the bile acid cholic acid. It rapidly and dramatically decreased expression of *Cyp17a1* mRNA and protein expression in liver (Figure 1C and D).

Next, to investigate the role of this receptor in the physiological regulation of hepatic Cyp17a1 by bile acids, mice lacking the bile acid sensor FXR [23] were used. During ad-libitum feeding, *Cyp17a1* expression was found to be significantly elevated in the livers of FXR^−/-^ mice compared to wild-type controls (Figure 1E). Thus, FXR physiologically suppresses hepatic Cyp17a1 during periods of nutrient availability. We corroborated our findings by showing that FXR was also required for the repressive effects of cholic-acid on Cyp17a1 expression (Figure 1E) This pattern of expression mirrors that of the prototypical indirect FXR target gene, *Cyp7a1* (Supplementary Fig. 1A and [10]). However, unlike *Cyp7a1* [24], the expression of *Cyp17a1* is not repressed by the gut-hormone FGF15/19 (Figure 1F). As such, hepatic *Cyp17a1* is likely to be regulated by a liver-autonomous FXR-signaling axis. Indeed, we found that the regulation of *Cyp17a1* by bile acids and FXR is liver-specific, as we saw no changes in expression in the adrenal or the ovary (Supplementary Fig. 1B. Taken together, these data demonstrate that bile acid signaling via FXR suppresses hepatic *Cyp17a1* expression, in a liver-autonomous manner, in the fed-state.

### 3.2 Nuclear receptors FXR, SHP, and LRH-1 regulate *Cyp17A1* promoter activity

**Figure 2:**
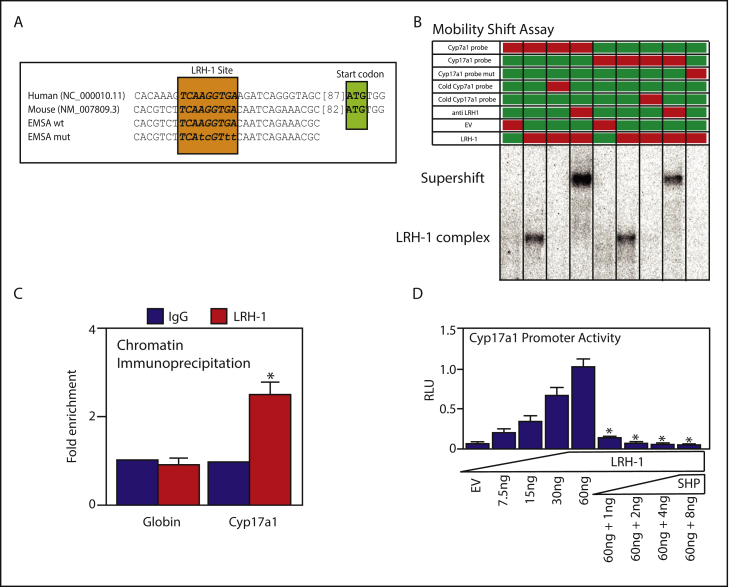
**The CYP17A1 promoter is a target of nuclear receptors LRH-1 and SHP.** (A) Predicted LRH-1 binding site in the mouse/human CYP17A1 promoter, and EMSA probes. (B and C) EMSA and ChIP assays demonstrating binding of LRH-1 to the CYP17A1 promoter. Red boxes indicate inclusion in the experiment. (D) Luciferase co-transfection assays showing that LRH-1 induces CYP17A1 transcription, which is repressed by the target of FXR, SHP. Data represent mean and SE of triplicates in an experiment conducted three times.

In the liver, FXR represses the rate-limiting enzyme in bile acid synthesis, *CYP7A1* through a nuclear receptor cascade. It induces the expression of *NR0B2* (*SHP*), which in turn inhibits LRH-1-mediated transcription of *CYP7A1* [10]. To begin to investigate whether *CYP17A1* expression is similarly regulated, we performed a search for potential LRH-1 binding sites in the *CYP17A1* promoter. A motif, containing only one mismatch with the consensus LRH-1 site, was identified at position −107 to −99bp upstream of the *CYP17A1* start codon (Figure 2A). LRH-1 was found to bind this site in mobility shift assays using the *CYP7A1* motif as a positive control (Figure 2B). The binding was specific because cold-probe competition completely prevented binding, co-incubation with LRH-1 antibodies super-shifted the complex, and mutation of the motif also prevented binding (Figure 2B). Finally, chromatin immunoprecipitation assays confirmed that LRH-1 also binds to the *CYP17a1* promoter in mouse liver (Figure 2C).

To ascertain whether LRH-1 binding to the *CYP17A1* promoter results in transcriptional activation, a *CYP17A1* reporter construct containing the LRH-1 binding site was cloned and co-transfected with increasing amounts of LRH-1 and/or SHP expression-plasmid. An identical experiment was conducted using the *CYP7a1*-promoter as a positive-control (Supplementary Fig. 2 and [10]). *CYP17A1* reporter activity increased in response to co-transfected LRH-1, and also decreased in response to co-transfected SHP (Figure 2D). These data identify *CYP17A1* as a novel molecular target of the FXR-signaling axis in liver in the post-prandial state.

### 3.3 *Cyp17a1* catalyzes the formation of DHEA, which activates the nutrient-sensor *PPARα*

**Figure 3:**
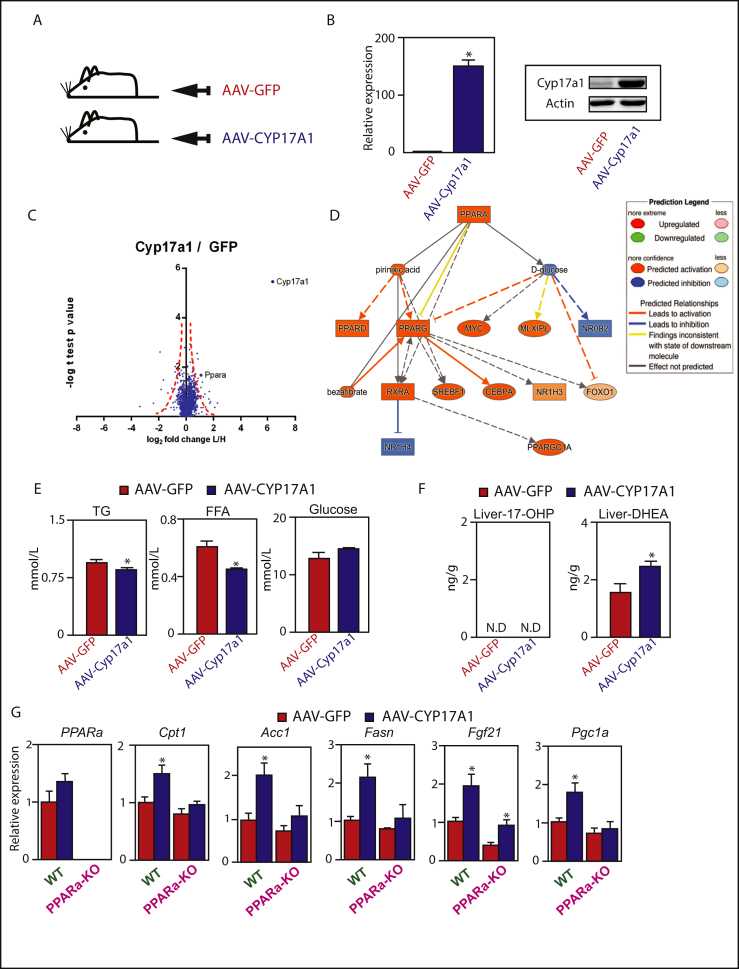
**Hepatic Cyp17a1 affects lipid handling by producing a ligand, DHEA, for PPARα.** (A and B) Forced expression of Cyp17a1 directed specifically to the liver and measured by qPCR and western blot. (C) Volcano plot of proteomic analysis in liver. (D) Ingenuity Pathway Analysis upstream regulator analysis demonstrating a state of PPARα activation in the Cyp17a1-overexpression group compared to the GFP-group. Solid lines depict direct relationships, dashed line depict indirect relationships. (E) Plasma parameters. (F) Cyp17a1 induces hepatic DHEA levels. (G) Metabolic mRNA expression in PPARα^−/-^ mice with and without forced expression of Cyp17a1 in liver. n = 5–6 *p < 0.05.

In order to investigate the function of Cyp17a1-activity in the liver, an AAV-mediated gain-of-function approach was undertaken to elevate the levels of the enzyme in the liver using a tissue-specific promoter (Figure 3A). Briefly, the mouse *Cyp17a1* gene was cloned downstream of the liver-specific LP1-promoter, packaged in to adeno-associated virus particles (AAV) that were injected into the tail vein of mice. Animals were sacrificed six-weeks after injection, and we confirmed that Cyp17a1 mRNA and protein expression was significantly increased in liver following AAV-Cyp17a1 injection (Figure 3B and Supplemental Fig. 3).

Subsequently, we conducted SILAC-based proteomics [25] on liver tissue. Briefly, liver protein extracts (containing ‘light’ lysine) were mixed 1:1 with a spike-in protein extract from ^13^C_6_-lysine metabolically labeled mouse liver (containing ‘heavy’ lysine) and analysed by LC-MS/MS. Significant expression differences between GFP-control and Cyp17a1-overexpression are depicted in a Volcano Plot (Figure 3C). As expected, Cyp17a1 represented the most significantly induced protein (Figure 3C and Supplementary Table 1). We subjected all proteins affected greater than 1.3-fold by Cyp17a1 over-expression to Upstream Regulator Analysis of the Ingenuity Pathway Analysis software package to predict upstream regulators of the changed protein signatures. By comparing the obtained differentially changed protein signatures and comparing them to the Ingenuity Knowledge Base, p-values were assigned based on significance of enrichment of the expression data for the proteins downstream of an upstream regulator. We found that the fasting-state nuclear receptor for fatty acids [7], PPARα, was the most significantly enriched *Upstream Regulator* in the Cyp17a1-overexpression group (Figure 3D).

In subsequent q-PCR analyses, we found that known lipid- and PPARα-regulated gene-transcripts were significantly induced in the livers of Cyp17a1 over-expressing animals. Indeed, we found statistically significant increases in the expression of a number of lipid-regulatory genes including those for fatty acid synthesis (*Fasn*), gluconeogenesis (*Pepck*), and metabolic regulation *Pgc1a*, *Fgf21* (p = 0.07)) (Supplementary Fig. 4A). These gene-expression changes, induced by over-expression of Cyp17a1 in the liver, also affected whole-body metabolic parameters. For example, plasma triglycerides and free fatty acids were decreased, and a trend towards elevated blood glucose levels was observed (Figure 3E). Cyp17a1 expression also increased plasma cholesterol, HDL, and LDL levels, but there was no effect on plasma insulin or circulating bile acids (Supplementary Fig. 4B). In summary, these data show that enhanced Cyp17a1-activity in the liver can drive changes in hepatic gene-expression that affect whole-body metabolic parameters.

Because Cyp17a1 may drive PPARα-activation, we hypothesized that its enzymatic activity catalyzed the production of a PPARα ligand. Therefore, we measured putative products of the Cyp17a1-reaction in liver extracts of AAV-Cyp17a1 mice. 17a-hydroxyprogesterone could not be detected using a commercially available ELISA (Figure 3F). However, Cyp17a1 is also known to generate dehydroepiandrosterone (DHEA), which has been shown to activate PPARα [26], and is elevated during fasting [13]. Indeed, DHEA was significantly increased in the livers of mice over-expressing Cyp17a1 (Figure 3F). We subsequently tested whether PPARα-activation specifically drives the metabolic effects of Cyp17a1-activity in the liver using PPARα^−/-^ mice [27]. Interestingly, despite our proteomic analysis detecting elevated levels of the PPARα protein, its mRNA expression was not changed (Figure 3G). This suggests that the product of the Cyp17a1 reaction in the liver may stabilize the PPARα protein, as has been described for other PPARα ligands [26]. In addition, we found that the Cyp17a1-mediated induction of multiple metabolic genes was indeed PPARα-dependent. Specifically, the Cyp17a1-mediated induction of hepatic *Cpt1*, *Acc1*, *Fasn*, and *Pgc1a* were all blunted in the absence of PPARα (Figure 3G). Together, these data demonstrate that hepatic Cyp17a1 produces a bio-active ligand for PPARα, DHEA, and drives expression of genes involved in glucose and lipid metabolic processes during fasting.

### 3.4 Fasting-induced *Cyp17a1* is essential for euglycemia and ketogenesis

**Figure 4:**
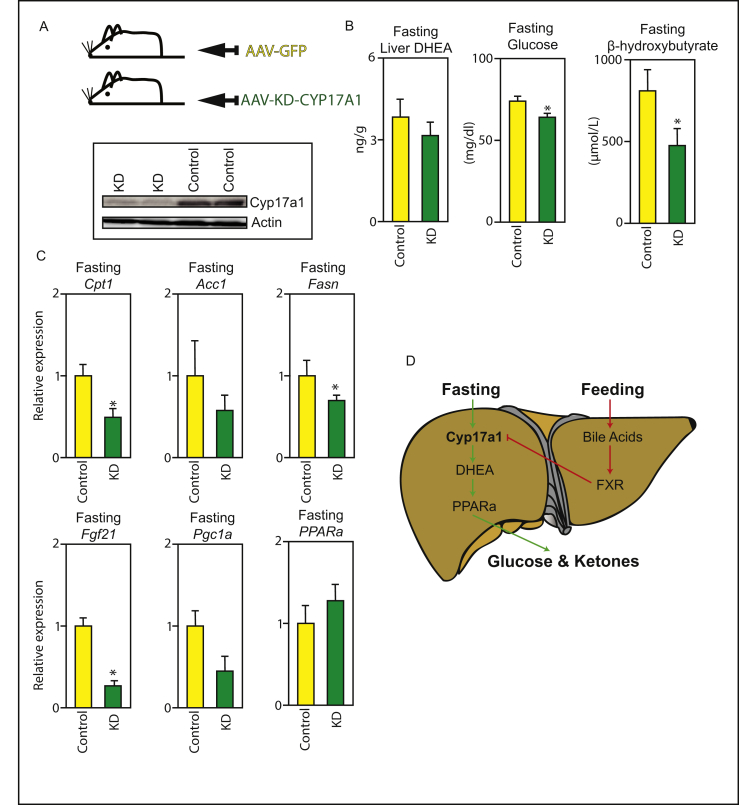
**Hepatic Cyp17a1 is essential for maintaining blood glucose ketone levels during fasting.** (A) Knockdown of Cyp17a1 specifically in the liver. (B and C) Fasting liver DHEA, blood-glucose, and β-hydroxybutyrate levels in mice fasted for 24 h with and without hepatic knockdown of Cyp17a1. (C) PPARα target-gene mRNA expression in fasted mice in the presence or absence of hepatic Cyp17a1. (D) Schematic summary of findings. n = 7–8, *p < 0.05.

In order to investigate the physiological relevance of hepatic Cyp17a1 activity during fasting, an AAV-mediated loss-of-function approach was undertaken to reduce the levels of the enzyme in the liver using a tissue-specific promoter (Figure 4A). Briefly, an shRNA designed to interfere with the *Cyp17a1*-transcript was cloned downstream of the liver-targeted AAV8-shCyp17a1, packaged in to adeno-associated virus particles (AAV) and injected into the tail vein. Animals were sacrificed six-weeks after injection, after 24 h fasting. The liver-targeted siRNA-AAV knockdown was confirmed by western blot (Figure 4A). Crucially, we found that mice fasted for 24 h with knockdown of hepatic Cyp17a1 had lower fasting glucose and β-hydroxybutyrate levels than control-injected mice (Figure 4B). As expected, plasma DHEA levels trended lower in the KD mice, although the analysis did not reach statistical significance (Figure 4B). This was accompanied by reduced expression of a number of PPARα target genes, including *Cpt1*, *Fasn*, and *Fgf21* (Figure 4C). *Acc1* and *Pgc1a* also trended lower (Figure 4C). This experiment demonstrates a critical role for hepatic Cyp17a1 as part of the adaptive starvation response in mice. Perturbing its activity blunts the induction of PPARα target-genes, and decreases systemic energy availability in the form of both glucose and ketones. These findings are consistent with an interpretation that extra-gonadal steroidogenesis by Cyp17a1, which becomes active during fasting due to reduced bile acid signaling, plays a key role in survival during nutrient-shortage by driving PPARα transcriptional-activity in the liver.

## 4 Discussion

In summary, we have shown that the classical steroidogenic enzyme Cyp17a1 is expressed in the liver and dramatically regulated by feed-fast cycles. It is suppressed in the fed-state through a bile-acid dependent nuclear receptor cascade involving repression of LRH-1 activity by FXR:SHP. Indeed, our promoter activity analyses are consistent with recent findings that LRH1 itself can also affect liver lipid levels [28] and that LRH-1 can transactivate steroidogenic enzymes in prostate cancer cells [29,30]. During the transition to a fasted-state, bile acid availability in the liver declines and hepatic *Cyp17a1* expression is de-repressed. During fasting it catalyzes the formation of at least one steroid-ligand, DHEA, which binds and activates the metabolic regulator PPARα (Figure 4D). While we saw the induction of some fatty acid synthesis genes in our overexpression experiment, and this may seem counterintuitive, these results are consistent with the finding that some SREBP target-genes are upregulated by PPARα-specific ligands [31,32].

Our loss-of-function data show that Cyp17a1 is an essential component of the adaptive starvation response. As such, these findings identify it as a crucial actuator in a model of adaptive liver physiology in which the nuclear receptors FXR and PPARα control anabolic and catabolic processes, respectively [6,33]. Our findings provide mechanistic and functional relevance to previous observations that hepatic *Cyp17a1* levels are higher in fasted animals [13], and explain the mechanism by which *Cyp17a1* was found to be elevated in mice lacking both FXR and SHP [5]. Our findings also represent a novel paradigm in which extra-gonadal steroidogenesis can feed-forward on to metabolic pathways to drive adaption to nutritional challenges. Finally, although governed physiologically by prandial changes in bile acids levels, the ability of hepatic Cyp17a1 to modify lipid and glucose-handling targets it for potential future intervention in metabolic disease.

## Author contributions

AM, VM, HV, IB, NA, HABP, EW, JN, BMO performed research. IMA, PB, SW, SV, WSD, SvM, BMO supervised research. AM, SvM, and BMO wrote the paper.

## Funding contributions

HV is supported by the Proteins at Work program of The Netherlands Organization for Scientific Research (NWO) (project number 184.032.201). NA is supported by a Rutherford Fund Fellowship. CW is funded by the Wellcome Trust (Grant P30874). WSD is funded by an NIHR Research Professorship. SvM is supported by the Netherlands Organization for Scientific Research (NWO) Project VIDI (917.11.365), FP7 Marie Curie Actions IAPP (FXR-IBD, 611979). BMO is supported by a Sir Henry Dale Fellowship jointly funded by The Wellcome Trust and The Royal Society (105545/Z/14/Z). This article presents independent research supported by the NIHR at Imperial College Healthcare NHS Trust. The Section of Endocrinology and Investigative Medicine is funded by grants from the MRC, BBSRC, NIHR, an Integrative Mammalian Biology (IMB) Capacity Building Award, an FP7- HEALTH- 2009- 241592 EuroCHIP grant and is supported by the NIHR Biomedical Research Centre Funding Scheme. The views expressed are those of the authors and not necessarily those of the funders, the NHS, the NIHR or the Department of Health.
