# Supplementary material for: Steroidogenic control of liver metabolism through a nuclear receptor-network
Source: Mol Metab. 2019 Sep 30;30:221–9. doi: 10.1016/j.molmet.2019.09.007 (PMC6819870; doi:10.1016/j.molmet.2019.09.007)
Supplement: Multimedia component 1 [file mmc1.pdf]

Supplemental Table 1

Gene Name	Fold Change
Cyp17a1	+83
Apo19b	+2.3
PPARa	+2.1
Hpd	+1.8
Gstm	+1.8
Me1	+1.8
Fabp2	+1.7
Cyp4a12	+1.6
Sdr39u1	+1.6
Acss2	+1.5
Herpud1	-1.5
Hsd17b6	-1.5
Cf1	-1.5
Cd44	-1.5
Tat	-1.7
Hmgcr	-1.7
Fgl1	-3.1
Tlcd2	-3.7
