# Supplementary figures and images for: Steroidogenic control of liver metabolism through a nuclear receptor-network
Source: Mol Metab. 2019 Sep 30;30:221–9. doi: 10.1016/j.molmet.2019.09.007 (PMC6819870; doi:10.1016/j.molmet.2019.09.007)

Supplemental Figure 1

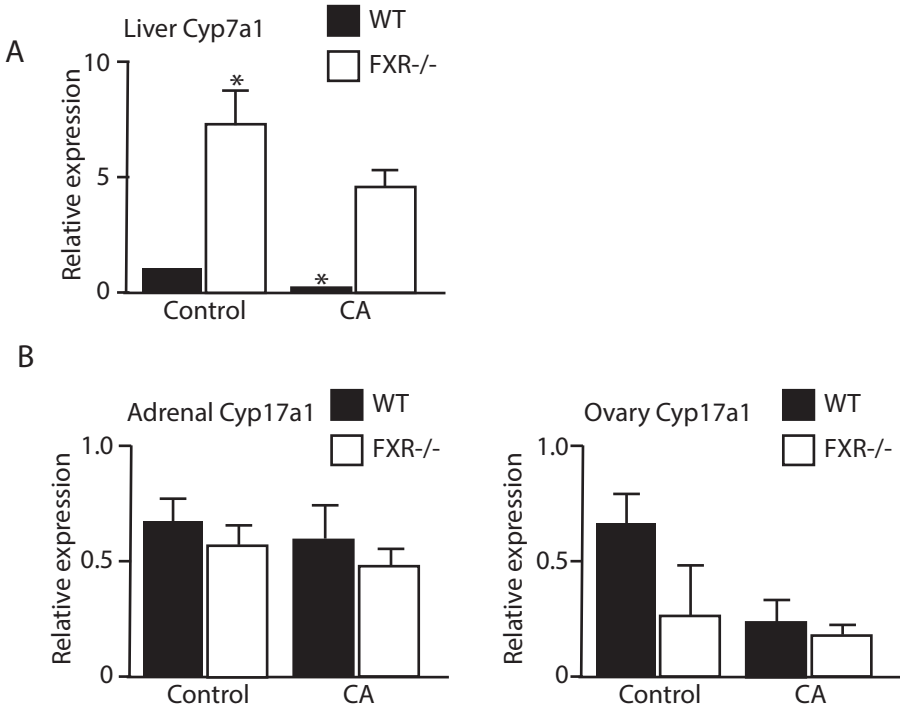

Supplement: Multimedia component 2 [file mmc2.pdf]

Supplemental Figure 2

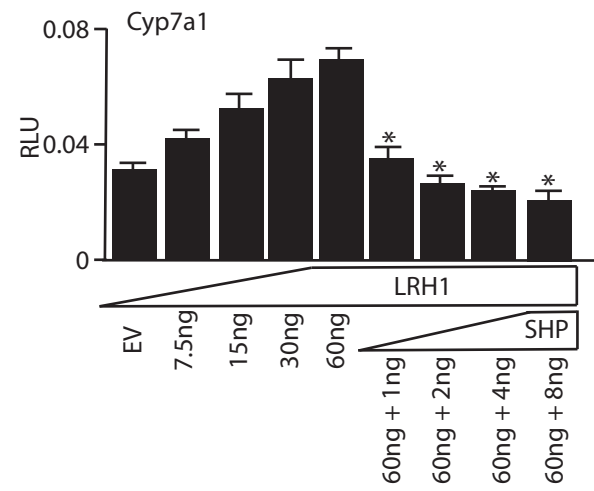

Supplement: Multimedia component 3 [file mmc3.pdf]

Supplemental Figure 2

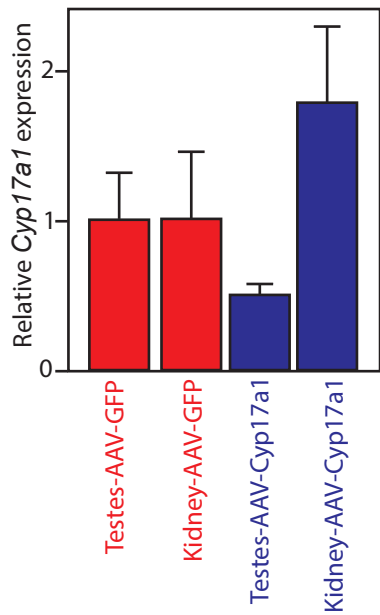

Supplement: Multimedia component 4 [file mmc4.pdf]

Supplemental Figure 4

A

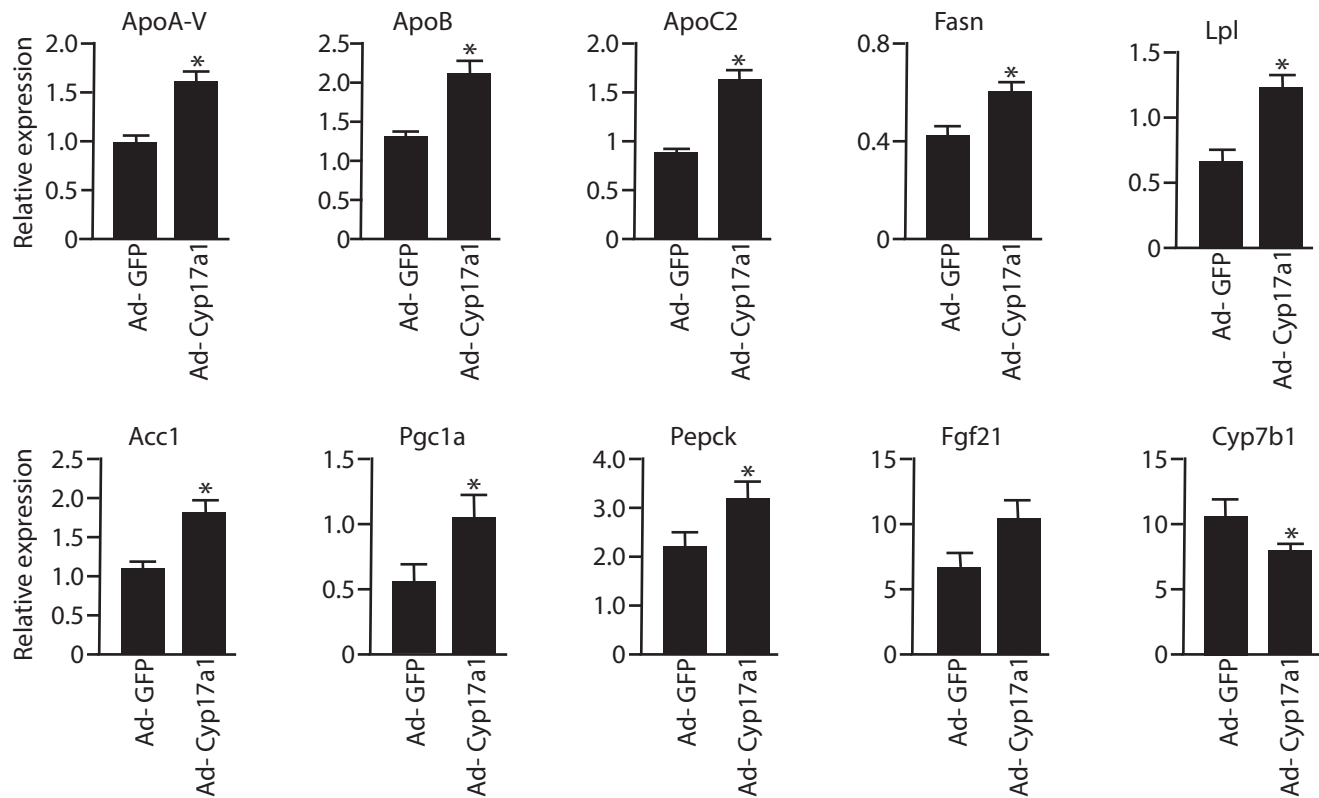

B

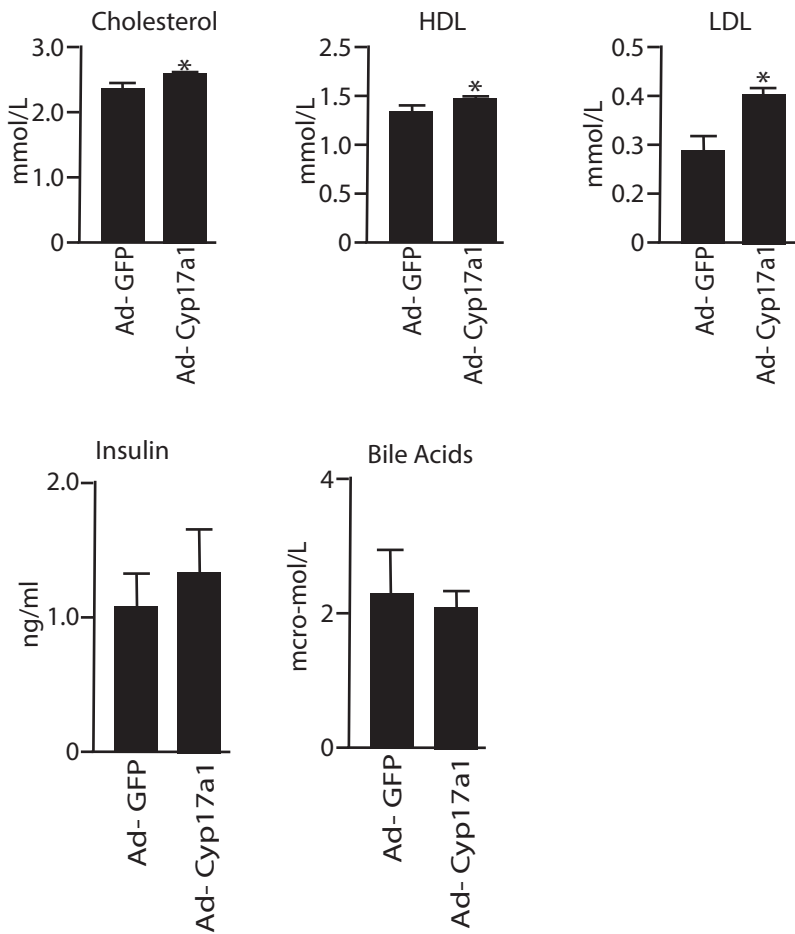

Supplement: Multimedia component 5 [file mmc5.pdf]
